# Chimeric antigen-receptor (CAR) engineered natural killer cells in a chronic myeloid leukemia (CML) blast crisis model

**DOI:** 10.3389/fimmu.2023.1309010

**Published:** 2024-01-08

**Authors:** Jusuf Imeri, Paul Marcoux, Matthias Huyghe, Christophe Desterke, Daianne Maciely Carvalho Fantacini, Frank Griscelli, Dimas T. Covas, Lucas Eduardo Botelho de Souza, Annelise Bennaceur Griscelli, Ali G. Turhan

**Affiliations:** ^1^ INSERM UMR-S-1310, Université Paris Saclay, Villejuif, France and ESTeam Paris Sud, Université Paris Saclay, Villejuif, France; ^2^ Blood Center of Ribeirão Preto/Ribeirão Preto School of Medicine/University of São Paulo, Ribeirao Preto, SP, Brazil; ^3^ INGESTEM National iPSC Infrastructure, Villejuif, France; ^4^ CITHERA, Centre for IPSC Therapies, INSERM UMS-45, Evry, France; ^5^ Université Paris Descartes, Faculté Sorbonne Paris Cité, Faculté des Sciences Pharmaceutiques et Biologiques, Paris, France; ^6^ Biotechnology Nucleus of Ribeirão Preto/Butantan Institute - Ribeirão Preto, Ribeirao Preto, SP, Brazil; ^7^ APHP Paris Saclay, Department of Hematology, Hopital Bicetre & Paul Brousse, Villejuif, France

**Keywords:** CAR-NK, NK92, CD25, leukemia, blast crisis CML

## Abstract

During the last two decades, the introduction of tyrosine kinase inhibitors (TKIs) to the therapy has changed the natural history of CML but progression into accelerated and blast phase (AP/BP) occurs in 3-5% of cases, especially in patients resistant to several lines of TKIs. In TKI-refractory patients in advanced phases, the only curative option is hematopoietic stem cell transplantation. We and others have shown the relevance of the expression of the Interleukin-2-Receptor α subunit (IL2RA/CD25) as a biomarker of CML progression, suggesting its potential use as a therapeutic target for CAR-based therapies. Here we show the development of a CAR-NK therapy model able to target efficiently a blast crisis cell line (K562). The design of the CAR was based on the scFv of the clinically approved anti-CD25 monoclonal antibody (Basiliximab). The CAR construct was integrated into NK92 cells resulting in the generation of CD25 CAR-NK92 cells. Target K562 cells were engineered by lentiviral gene transfer of CD25. *In vitro* functionality experiments and *in vivo* leukemogenicity experiments in NSG mice transplanted by K562-CD25 cells showed the efficacy and specificity of this strategy. These proof-of-concept studies could represent a first step for further development of this technology in refractory/relapsed (R/R) CML patients in BP as well as in R/R acute myeloblastic leukemias (AML).

## 1 Introduction

Chronic myeloid leukemia (CML) is a clonal hematopoietic stem cell disorder characterized by the reciprocal translocation t(9;22) (q34;q11.2) (1). As a result, a *BCR::ABL* hybrid gene is formed on the derivative Ph chromosome and is characterized by a dysregulated tyrosine kinase (TK) activity (2, 3). The use of tyrosine kinase inhibitors (TKIs) has dramatically modified the therapy of CML, generating durable remissions and prolonging survival in TKI responders. TKI therapies allow, albeit with different efficiencies, deep molecular responses in the majority of patients but TKI-cessation attempts show that despite these long-lasting deep responses, rapid molecular recurrence occurs in approximately 50% of cases. The highly quiescent leukemic stem cells (LSCs) are known to be resistant to TKIs (4, 5) and responsible for relapse after TKI treatment cessation (6–9). Therefore, most patients require the administration of life-long TKI therapies and a large fraction develop resistance to these drugs. TKI resistance and disease progression into an advanced phase/blast phase (AP/BP) remains problematic especially in this last group of patients as the only curative approach remains stem cell transplantation. This therapy is difficult to apply in patients lacking donors and especially in patients with significant co-morbidities. Hence, the development of new therapies based on targets expressed in AP/BP CML remains an unmet medical need even in the era of TKI therapies.

CD25 (also known as the α subunit of IL2R) is one of the three subunits that constitute the receptor to IL-2, the two others are the β and the γ subunit or CD122 and CD132 respectively (10). In normal conditions, CD25 is highly expressed in activated T cells and regulatory T cells, which plays an important role in the homeostasis of T cell activity and immune tolerance (11, 12). CD25 is also shown to be expressed in, BP-CML, BP-CML transformed in acute myeloid leukemia (AML) but also in *de novo* AML with dismal prognosis (13–19). More interestingly, CD25 is shown to be aberrantly expressed in the LSC of CML rendering it a very interesting target for the eradication of the LSC and the CML treatment (20).

We have previously shown that CD25 is overexpressed in BP-CML model generated from patient-specific induced pluripotent stem cells (iPSC) and confirmed the increase of its expression during CML progression (21). These findings prompted us to evaluate the possibility of targeting this receptor using chimeric antigen receptor (CAR)-based therapy in the setting of CML.

CARs are fusion proteins introduced into lymphocytes (T and NK) and recently into macrophages to confer them specific recognition skills. During the last decade, cell therapies based on CAR-T technology have shown groundbreaking results in R/R hematological malignancies leading to their clinical licensing in acute lymphoblastic leukemia (ALL) and lymphomas, by essentially targeting the B-cell marker CD19 (22). Recently, CAR-NK has been introduced as a solution to the limitations of allogeneic transplants which are associated with side effects such as graft versus host disease (GvHD) of CAR-T cells (23, 24). In the context of CML, the use of CAR-NK cells has a double interest because NK cells are known to have also a CAR-independent anti-leukemic effect inherent to their antitumoral properties (25, 26).

Here we show the experimental development of a CAR-NK therapy strategy against the IL2RA/CD25 which as described above has been previously reported as being overexpressed, in advanced CML (21). This strategy is based on the use of a single chain variable fragment (scFv) of the clinically approved monoclonal humanized antibody, Basiliximab recognizing specifically CD25 and targeting K562 cells that we have genetically engineered to overexpress CD25. We therefore use the intrinsic antileukemic potential of NK92 cells and the increased specificity and activation through the CAR constructs to target K562-CD25 cells. We demonstrate an increased cytotoxic activity of CD25 CAR-NK92 cells against leukemic cells expressing CD25 and their efficiency *in vivo*

## 2 Materials and methods

### 2.1 Cell culture

The Lenti-X 293T cell line (a kind gift from Dr. Lucas Botelho de Souza) was cultured in Dulbecco’s Modified Eagle’s Medium (DMEM) supplemented with 10% fetal bovine serum (FBS) (Gibco, 11560636) and 100 U/mL of penicillin-streptomycin (PenStrep) solution (Gibco, 11548876). K562 were cultured in RPMI 1640 (Gibco, 11875093) supplemented with 10% of FBS and 100 U/mL of PenStrep. NK92 were cultured in RPMI 1640 supplemented with 20% of FBS, 100 U/mL of PenStrep, and 150 U/mL of IL-2 (Miltenyi Biotec, 130-097-745). NK92 and K562 cells were passaged every 3 to 4 days at a dilution of 3x10^5^ cells/mL and Lentix were plated at 3.5x10^4^/cm^2^.

### 2.2 Lentiviral production and viral transduction

In order to produce IL2RA expressing lentiviruses, we used Lenti-X 293T as a packaging cell line and ps-PAX2.2, and pMD2.G as packaging vector and envelope vector, respectively. Briefly, the Lentix-293T cells were cultured in a T150 mm dish as described above and co-transfected by lipofectamine 3000 reagent (ThermoFisher, L3000015) with 20μg packaging vector of ps-PAX2.2 (Addgene, USA), 10μg envelope vector of pMD2.G (Addgene, USA) and 30μg transfer vector (EF1a-hIL2RA). The supernatant was collected at 24h and 48h. K562 cells were transduced by the use of unconcentrated virus at three different dilutions. Transduced cells were spinoculated at 1200 rpm for 90 minutes at RT and 8 μg/mL of Polybrene was added. After 48 hours, the virus was removed and transduced cells were selected with 5μg/mL Blastcidine S (Gibco, A1113903). Resistant cells were analyzed by FACS and sorted for CD25.

For CD25 CAR lentivirus production, Lentix-293T cells have been transfected with 3^rd^ generation lentiviral system using lipofectamine 3000 with the following plasmids: pLP1 (HIV-1 gag and pol), pLP2 (HIV-1 rev), pLP VSV-G (vesicular stomatitis virus G glycoprotein) and the backbone with the following ratios 5: 5: 3: 7. Transduction was performed as described above.

### 2.3 Molecular constructs

**Figure 1:**
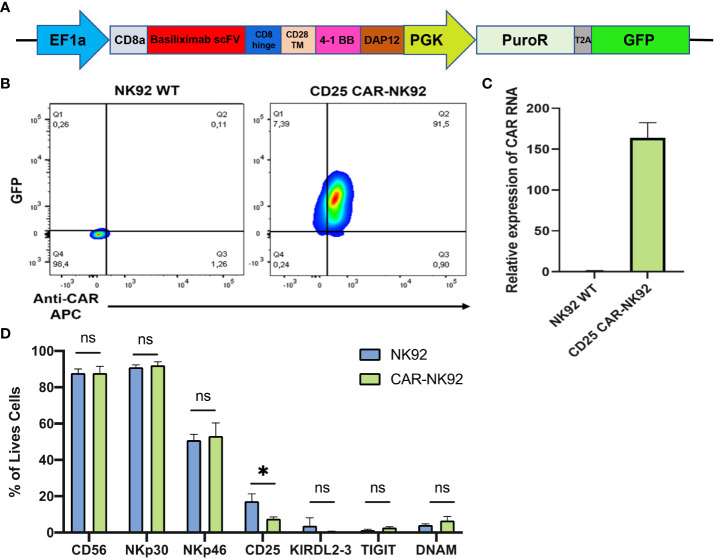
Generation of CAR-NK cells from NK92 cells. **(A)** Constitutive CAR expression construct under EF1a promoter followed by puromycin resistance and GFP under PGK promoter. **(B)** CAR and GFP expression level of NK92 transduced cells gated on NK92 WT. **(C, D)** Main NK markers expressed on CAR-NK cells (in pink) and in NK-92 WT cells (in blue). Means and SD are represented. Experiments have been performed 3 times. P-values were calculated using a 2-tailed Student’s t-test. ns, not significant; *P < 0.05.

The EF1a-hIL2RA lentivirus plasmid was purchased from VectorBuilder (Guangzhou, China). CD25 CAR lentivirus expression plasmids were purchased from Creative Biolabs (New York, USA). In this construct, Basiliximab scFv was used under EF1a promoter followed by the CD8a hinge domain, CD28 transmembrane domain, 4-1BB co-stimulatory domain, and DAP12 for NK cell activation. GFP and Puromycin resistance were used as selection markers (**Figure 1A**).

### 2.4 RNA extraction and qRT-PCR

Total RNA was extracted using RNeasy Mini Kit (74104; Qiagen, Germany) and 1 µg was reverse transcribed using a reverse transcription (RT)-PCR kit (Superscript III 18080-44; ThermoFisher Scientific). An aliquot of cDNA was used as a template for qRT-PCR analysis using a fluorescence thermocycler (ThermoFisher Scientific QuantStudio 3TM) with FastStart Universal SYBR Green (04913914001, Roche) DNA dye. For the amplification of the housekeeping gene (Actin B), the following primers have been used, Forward: “GTGGCCGAGGACTTTGATTG” and Reverse: “TGGACTTGGGAGAAGGACTGG” and for the CAR sequence, Forward: “GGCCCCAGAAGTCGAAGTAG” and Reverse: “ACCAGAAGTTCGAGGGCAAG”. Relative expression was normalized to the geometric mean of housekeeping gene expression and was calculated using the 2^-ΔΔCt^ method.

### 2.5 Flow cytometry

For extracellular staining, cells were washed with DPBS and incubated with antibodies in a volume of 100 μL of DPBS with an adequate amount of antibody (**Table 1**). For intracellular staining, cells were thereafter fixed with BD Cytofix™ (BD, 554655) for 10 minutes at room temperature, protected from light, washed, and permeabilized with BD Perm/Wash™ (BD, 555028) together with intracellular antibodies for 20 minutes at 4°C. Flow cytometry was performed with a BD FACS LSRFortessa™ and data were analyzed using FlowJo.

**Table 1:** Antibodies used for FACS and their respective references.

*Antibody and Clone*	*Reference*
AffiniPure F(AB) Anti-mouse IgG Alexa Fluor 647 Polyclonal	115-606-072-Jackson ImmunoResearch
CD56-APC B159	555518-Becton Dickinson
CD25-APC REA945	130-115-535 Miltenyi Biotec
CD107a-PE-Vio 770 REA792	130-111-622 - Miltenyi Biotec
Granzyme B-BV421 GB11	563389-Becton Dickinson
KIRDL2/3-PE-Vio 770 REA1006	130-116-835-Miltenyi Biotec
NKp30-PE-Vio 770 RE823	130-112-432-Miltenyi Biotec
NKp46-PE-Vio 770 REA808	130-112-123-Miltenyi Biotec
Perforin-BV421 δG9	563393-Becton Dickinson
TIGIT-PE-Vio 770 REA1004	130-116-817-Miltenyi Biotec

### 2.6 Cell sorting

Cells were stained with extracellular antibodies as described above and were resuspended in their culture media at a concentration of 5x10^6^ cells/mL. Thereafter, they were sorted with a BD FACSAria™ SORP (BD) and sorted cells were immediately resuspended at the adequate concentration and cultured at 37°C, 5% CO_2_.

### 2.7 CD107a expression and IFNγ expression

Wild type NK92 (NK92 WT) and CD25 CAR-NK92 cells were co-cultured with target cells in a 96 well plate. Antibody was added to each well at a dilution of 1:100 and incubated for 1 hour. Golgistop and GolgiPlug (554724 and 555029, BD) were thereafter added for 2 additional hours. After the incubation cells were washed with FACS buffer and stained with CD56 surface marker for 25 minutes at 4°C protected from light. Following this last incubation, cells were washed with FACS buffer and analyzed by FACS.

### 2.8 Annexin V apoptosis assay

Apoptosis induced by NK cells was evaluated by staining Annexin V on the surface of target cells previously stained with CellTrace™ Yellow (C34567, ThermoFisher Scientfic). For CellTrace staining, cells were incubated with 1 μL of the dye for 1x10^6^ cells/mL in PBS for 20 min at 37°C. Thereafter, the free dye was removed by adding five times the original staining volume media containing 10% FBS. After 2h of co-culture as described above, cells were stained for Annexin V according to the manufacturer’s instruction. Briefly, cells were washed with 1X binding buffer, centrifuged and resuspended in binding buffer containing AnnexinV-APC (88-8007-74, ThermoFisher Scientific). After 15 minutes incubation, cells were washed again and analyzed by FACS.

### 2.9 *In vivo* assays

NSG (NOD-Prkdc^scid^ IL2rg^tm1^/Bcgen) mice were intraperitoneally injected with K562-CD25 cells expressing Luciferase at Day -3 (3x10^6^ cells/mouse, n = 30). At Days 0, 3, and 7, mice were injected intraperitoneally with either 10 Gy irradiated CD25 CAR-NK92 cells (10x10^6^/mouse; n=10) or irradiated wild-type NK92 cells (n=10). The clinical evolution of transplanted mice was followed by luminescence (IVIS 200, Caliper Life Sciences).

### 2.10 Statistical analysis

Two-tailed Student’s t-tests were performed using GraphPad Prism version 10.0.0 for Windows, GraphPad Software, Boston, Massachusetts USA, www.graphpad.com. Means and SD are represented on the graphs. All experiments have been performed 3 times. ns, not significant; *, P < 0.05; **, P < 0.01; ***, P < 0.001; ****, P < 0.0001. Survival analyses were performed in R software environment version 4.2.1. Overall survival stratified on experimental groups was fitted with survival R-package version 3.5-7. Kaplan-Meier plot and log-rank test analysis were done with survminer R-package version 0.4.9.

## 3 Results

### 3.1 Generation of K562 cells overexpressing ILRA/CD25

**Figure 2:**
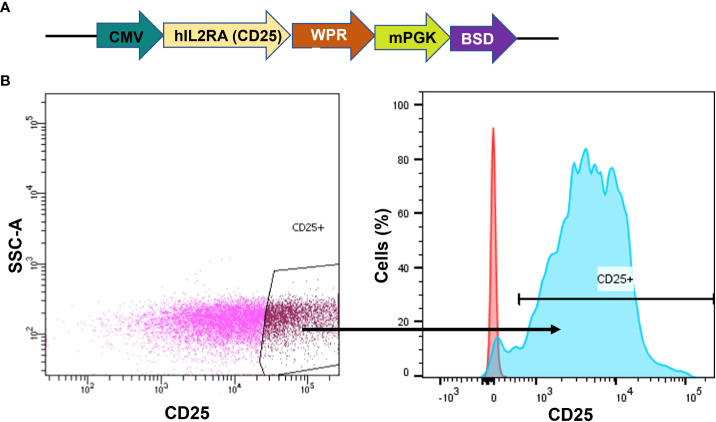
Generation of K562 cells expressing CD25. **(A)** hCD25 expression vector under the control of CMV promoter and followed by Blasticidin S resistance. **(B)** CD25 expression on K562 cell line after lentiviral transduction. The gate shows the sorted population (left). CD25 expression on sorted cells (in blue) as compared to K562 WT (in red) (right).

As the native K562 cells lack CD25 receptor expression, our initial step involved generating a CD25 positive cell line to serve as a proof-of-concept for our CAR-CD25 strategy. Therefore, a CD25 transgene was introduced into K562 leukemic cells via lentiviral transduction. The CD25 gene was under the control of the CMV promoter and Blasticidin S resistance was used as a selection marker (**Figure 2A**). Following antibiotic selection and CD25^+^ cell sorting, we successfully acquired a highly purified population with CD25 expression levels approaching 100% (K562-CD25) (**Figure 2B**, blue). Therefore, these cells serve as an ideal CML model expressing CD25, making them the optimal choice for assessing the effectiveness of CD25 CAR-NK92 cells.

### 3.2 Generation of NK92 cells expressing CD25 CAR

The anti-CD25 CAR-NK was designed as a polycistronic construct, incorporating the scFv from the clinically approved monoclonal antibody Basiliximab, driven by the potent EF1a promoter. CD8a was employed as a signal peptide to address the CAR protein to the cell surface. Two costimulatory domains, 4-1BB as well as the NK-specific DAP12 were incorporated, followed by puromycin resistance and GFP, with both genes being regulated by the PGK promoter (**Figure 1A**). Following lentiviral transduction of NK92 cells with the CAR construct, transduced cells were evaluated based on their GFP expression and the level of CAR expression at their cell surface. Approximately 90% of cells were found to be double-positive (GFP^+^ and CAR^+^) while no GFP or CAR expression was detected in NK92 WT (**Figure 1B**). The level of CAR expression was also evaluated by qRT-PCR and we observed a 150-fold higher relative expression of CAR mRNA in CD25 CAR-NK92 cells as compared to untransduced cells (**Figure 1C**). In terms of phenotype, there was no significant distinction in the expression of NK activating and inhibitory signals between CD25 CAR-NK92 and NK92 WT. This suggest that the transduction did not affect the phenotype of the cells. Intriguingly, we observed a reduction in the level of CD25 expression in CD25 CAR-NK92 when compared to NK92 WT (**Figure 1D**).

### 3.3 CD25 CAR-NK92 cells exhibit a higher activated profile and cytotoxicity as compared to NK92 WT

**Figure 3:**
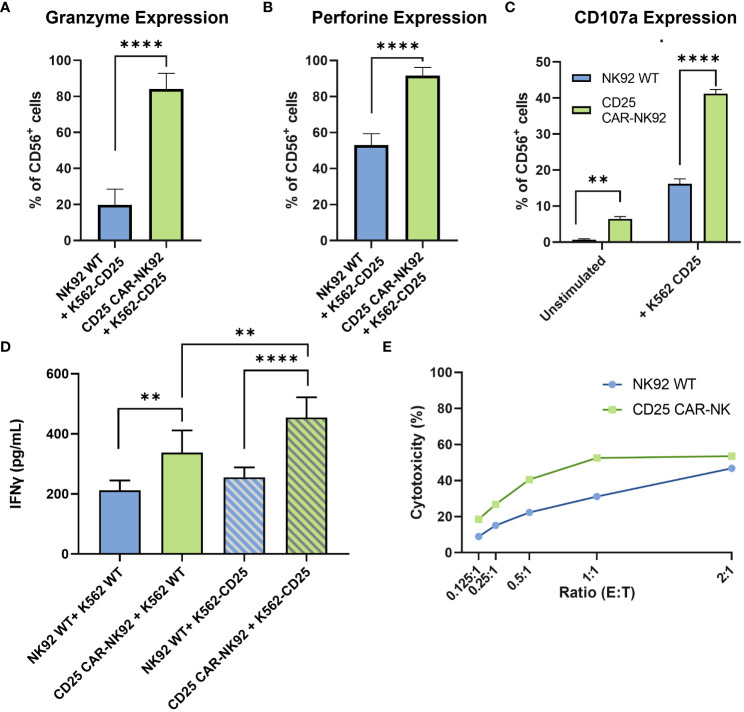
Functional and cytotoxic assessment of CD25 CAR-NK92 cells. **(A, B)** Granzyme B and Perforin expression levels of CD25 CAR-NK92 and NK92 WT cells after co-culture with K562-CD25 cells. **(C)** Degranulation assay of NK92 WT and CD25 CAR-NK92 cells after co-culture with K562-CD25. **(D)** IFNγ levels assessment of CD25 CAR-NK92 and NK92 WT after stimulation either with K562 WT or K562-CD25. **(E)** Cytotoxic assay at different Effector (E) Tumor (T) ratios for NK92 WT: K562-CD25 in blue and CD25 CAR-NK92 in green. Means and SD are represented. Experiments have been performed 3 times. P-values were calculated using a 2-tailed Student’s t-test. **P < 0.01; ****P < 0.0001.

In order to assess the functionality of the CD25 CAR-NK92 cells, we evaluated the level of Granzyme B and Perforin expression following their co-cultivation with K562-CD25 cells. We observed a remarkable increase in Granzyme B expression in CD25 CAR-NK92 cells as compared to NK92 WT (**Figure 3A**). The levels of Perforin were assessed in the same conditions and were found to be significantly higher in CD25 CAR-NK92 cells as compared to NK92 WT (**Figure 3B**).

The degranulation potential was assessed by CD107a staining of NK92 WT and CD25 CAR-NK92 cells cocultured with or without K562-CD25. As can be observed in **Figure 3C**, CD25 CAR-NK92 cells show a much higher degranulation profile as compared to NK92 WT when stimulated with K562-CD25 (**Figure 3C**).

IFNγ production after stimulation of CD25 CAR-NK92 and NK92 WT was assessed by ELISA. We observed a strong increase of IFNγ levels in the CD25 CAR-NK92 condition co-cultured with K562-CD25 as compared to NK92 WT (p<0.0001) (**Figure 3D**). This suggests a much stronger activation of CD25 CAR-NK92 cells when co-cultured with K562-CD25 as compared to NK92 WT co-cultured with K562-CD25. We also observed a statistical difference (p<0.01) between the levels of IFNγ produced by CD25 CAR-NK92 and NK92 WT when co-cultured with K562-WT showing a higher inherent activity of CAR cells as compared to WT (**Figure 3D**).

In order to evaluate the cytotoxic potential of CD25 CAR-NK92 cells, the number of apoptotic cells resulting from a co-culture with K562-CD25 at different ratios was assessed. We observed a much higher percentage of apoptotic K562-CD25 cells (Annexin V^+^) in the CD25 CAR-NK92 condition as compared to NK92 WT for all ratios. This is coherent with the high level of activation of CD25 CAR-NK92 cells observed above (**Figure 3E**).

### 3.4 CD25 CAR-NK92 cells show enhanced *in vivo* antitumoral effect against K562-CD25 as compared to NK92 WT cells

**Figure 4:**
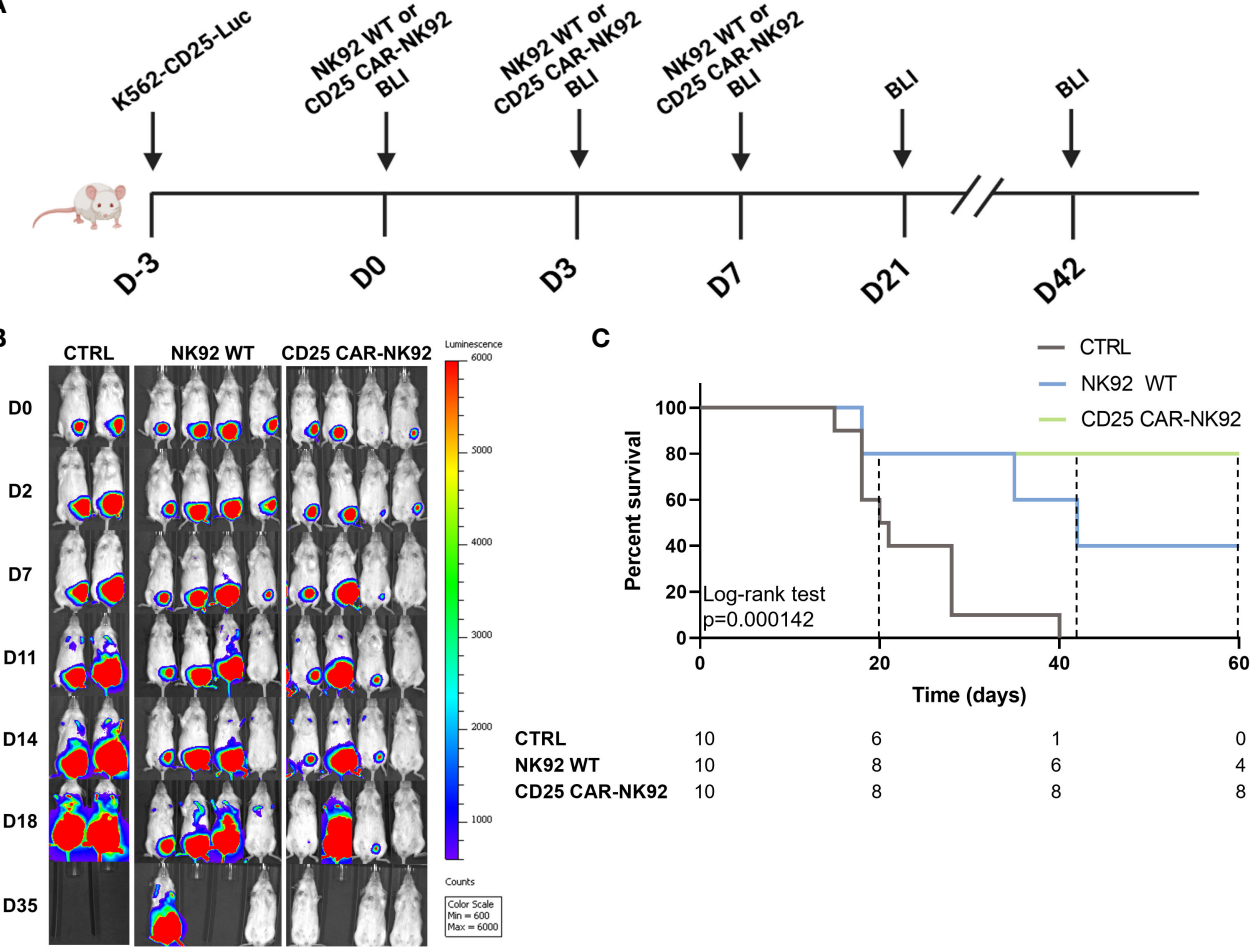
*In vivo* antitumoral activity assessment of CD25 CAR-NK92 cells. **(A)** Schematic representation of the experimental procedure, (BLI – Bioluminescence imaging) **(B)** BLI obtained by IVIS of three groups of mice, untreated (CTRL), NK92 WT treated in the middle and CD25 CAR-NK92 treated at the left **(C)** Kaplan-Meir and log-rank p-value of the overall survival stratified on experimental groups. survival curve of the three groups.

We next assessed the anti-leukemic activity of CD25 CAR-NK92 *in vivo* by using a noncurative mouse model of K562-CD25 cells. NSG (NOD-Prkdc^scid^ IL2rg^tm1^/Bcgen) mice were injected intraperitoneally at D-3 with K562-CD25 Luciferin expressing (Luc) leukemic cells. The mice were treated by the injection of either NK92 WT or CD25 CAR-NK92 at D0, D3 and D7. Mice were followed for two months and imaged regularly by bioluminescence (BLI) (**Figure 4A**). As shown in **Figure 4B**, untreated mice exhibited a fast-developing cancer, mostly isolated at the beginning but later spreading in the peritoneum. Mice treated with NK92 WT cells had a delayed development of leukemia as compared to the untreated cohort which is related to the natural antileukemic activity of NK92 cells. On the other hand, the cohort treated with CD25 CAR-NK92 cells showed much slower cancer progression resulting in mice that were completely tumor-free after one week of treatment (**Figure 4B**). This result is shown with the Kaplan-Meier curve in **Figure 4C**, in which the efficiency of CD25 CAR-NK92 therapy appears clearly significant in the survival rate as compared to the untreated and NK92 WT treated cohorts. Indeed, the majority of untreated mice died between D20 and D30. In the NK92 WT treated cohort, 6 mice survived at D40 which is coherent with the delay due to the NK92 antileukemic activity observed also previously. At day 60, only 4 mice were still alive in the NK92 WT treated cohort. While in the CD25 CAR-NK92 condition, two mice died around D20 and the others remained tumor-free without rejection for more than two months (**Figure 4C**). This shows the major interest of targeted therapy via CD25 CAR-NK92 cells against K562 cells expressing CD25, validating the potential of this strategy for future clinical use in blast-phase CML and AML.

## 4 Discussion

Although the use of TKI therapies against CML has allowed tremendous progress during the past twenty years, the development of TKI resistance and CML progression remains still a challenging issue because of limited therapeutic solutions at this stage of the disease. Developing new therapeutic tools against AP/BP-CML is therefore an unmet medical need in this life-threatening step of CML. We and others have shown previously the involvement of CD25 as a biomarker detected in primary BP-CML (14, 20) as well as its expression in *in vitro* BP-CML models (21). These findings prompted us to design CAR-NK to target CD25 as an off—the—shelf immunotherapy for CML blast crisis. The advantages of NK cells over T cells for CAR-based therapies are multiple. Firstly, NK cells have the innate ability to kill cancer cells or virally infected cells (27). Additionally, they have the advantage to induce cytotoxicity in an HLA-independent manner and do not give rise to graft-versus-host-disease (GvHD) which is the major therapeutic issue in the setting of allogeneic stem cell transplantation. Other advantages of NK cells over T cells include the absence of cytokine release syndrome or neurotoxicity (23, 24), as well as their antibody-dependent cellular cytotoxicity (ADCC) and their ability to kill tumor cells in a CAR-independent manner (25). The advantage of using a CAR-based strategy over existing monoclonal antibodies (mAb) is the fact that CAR-NK and CAR-T cells directly lyse the tumor cell upon engaging the antigen using the physiologic cytotoxic machinery of killer cells (28). Moreover, CAR cells have the ability to target cells with low antigen expression which can escape to mAb (28). In the context of CML progression, it has been shown that NK cells have a central role in the control of the disease and the anti-leukemic activity of NK cells inversely correlates to disease progression (29). For that reason, restoring the activity of NK cells in CML patients has been widely studied and the CAR-NK approach is of particular interest because it combines specificity and functional NK activity (29).

Basiliximab is a chimeric mouse-human monoclonal antibody to the α chain of the IL-2 receptor. It is indicated for the prophylaxis of acute organ rejection in *de novo* allogeneic renal transplantation (30). To the best of our knowledge, its scFv domain has never been used against CD25 in anti-tumoral approaches. The other anti-CD25 monoclonal antibody, Daclizumab, has been withdrawn from the market.

In order to generate CD25 CAR-NK92, we transduced the NK92 cell line with CAR-expressing lentivector. Phenotypic analyses showed no loss of the main NK markers. Interestingly, the loss of CD25 expression in the CD25 CAR-NK92 condition was not related to fratricide because no increased apoptosis was observed (Data not shown). The levels of Granzyme B and Perforin after stimulation with K562-CD25 cells in CD25 CAR-NK92, were coherent with the increased activity and degranulation of these cells. Consequently, we observed higher levels of IFNγ and apoptosis *in vitro*. *In vivo* assay showed a clear difference between the NK92 WT and CD25 CAR-NK92 where tumor-free mice survived without rejection for more than three months.

NK92 cells have an inherent anti-leukemic activity against K562 (31) and this is observed in all experiments with K562-CD25. Nevertheless, the CD25 CAR introduction conferred an increased antileukemic activity against K562-CD25 cells.

Although CAR-NK therapies exhibit a major clinical potential due to their safer profile in terms of GvHD as compared to CAR-T cells as well as to their HLA independence and allogenic utility, some questions remain still to be raised. One of these is the source of NK cells for therapy. Heterogeneity and batch-to-batch differences for peripheral blood-NK (PB-NK) and umbilical cord blood (UCB-NK) as well as the accessibility suggest the need for new NK sources. NK92 cell line, even though largely used in therapy after irradiation, remains a tumorigenic cell line with potential risks for the patients.

Immunotherapies based mostly on CAR-T and more recently in CAR-NK cells against myeloid malignancies especially AML are giving hope for the treatment of these diseases (32). In BP-CML, recent work has shown the feasibility of targeting CD38^+^ blast cells using autologous CAR-T cells (33). The interest of a CD25 CAR-NK approach among existing CAR-T therapies against myeloid malignancies is the combination of the specific targeting (CD25) of blast cells with NK cells whose advantages over T cells are described above. Moreover, CD25 is also shown to be expressed in the leukemic stem cell compartment of CML, suggesting an additional potential beneficial effect of this approach for the eradication of CML stem cells.

It would be also of great interest to associate Ponatinib with the CD25 targeting approach by CAR-NK strategy. Indeed, Ponatinib is used alone or in combination with other agents in patients with BP-CML (34) but relapses and or resistance are common with dismal prognosis in the absence of stem cell transplantation.

In healthy adults, the CD25 molecule is expressed in regulatory T cells (Tregs) playing a crucial role in the maintenance of immunological self-tolerance (35). These cells function throughout life by suppressing the activity of autoreactive T cells and represent therefore a major cell population against the development of autoimmunity (36). Tregs have also been shown to play a role in cancer and the depletion of Tregs could be beneficial in cancer therapy (37). Interestingly, numbers and frequencies of Tregs in peripheral blood and bone marrow are increased in CML patients at diagnosis (38–40). Moreover, Tregs are shown to be involved in the immune escape of leukemic stem cells (41) and the depletion of CD25^+^ Tregs has been shown to enhance effector activation and antitumor immunity (42). Therefore, a potential depletion of this increased Treg population in CML by CD25 CAR-NK92 might be of interest.

As opposed to CAR-T cells the adverse effects of which are well-established (43) CAR-NK cells are expected to have less toxicity with a high level of GVL effect and the absence of GVHD effects (44). Further experiments are needed to determine the off-side activity and the ability of CD25 CAR-NK92 cells to target non-cancerous CD25-expressing cells. The determination of the expression of stress markers in Tregs and other tissues would be important to predict potential off-target activity.

Altogether, these results pave the way for a new potential way of fighting BP-CML by targeting together the leukemic blast cell mass as well as LSCs, while potentially inhibiting by the same approach the immune escape of the leukemic cells.

## Data availability statement

The original contributions presented in the study are included in the article/supplementary material. Further inquiries can be directed to the corresponding author.

## Ethics statement

The animal study was approved by Inserm Animal Ethical Committee. The study was conducted in accordance with the local legislation and institutional requirements.

## Author contributions

JI: Conceptualization, Investigation, Writing – original draft, Writing – review & editing, Formal analysis, Methodology. PM: Formal analysis, Investigation, Methodology, Writing – review & editing. MH: Investigation, Methodology, Writing – review & editing. CD: Data curation, Investigation, Methodology, Validation, Writing – review & editing. DMC: Investigation, Methodology, Writing – review & editing. FG: Investigation, Resources, Supervision, Writing – review & editing. DTC: Resources, Supervision, Writing – review & editing, Project administration. LD: Investigation, Methodology, Conceptualization, Data curation, Resources, Validation, Writing – review & editing. AB: Resources, Supervision, Conceptualization, Investigation, Writing – review & editing. AT: Investigation, Resources, Supervision, Conceptualization, Project administration, Writing – original draft, Writing – review & editing.
